# Dysphasia and Phantosmia as First Presentation of Multifocal Cerebral Anaplastic Astrocytomas: Case Report and Review of the Literatures

**DOI:** 10.1097/MD.0000000000000877

**Published:** 2015-05-22

**Authors:** Xiangyi Kong, Yu Wang, Shuai Liu, Zhaohui Lu, Huanwen Wu, Xinxin Mao, Xin Cheng, Jun Gao, Jian Guan, Yi Yang, Yongning Li, Bing Xing, Wenbin Ma, Renzhi Wang

**Affiliations:** From the Departments of Neurosurgery (XK, YW, SL, JG, JG, YY, YL, BX, WM, RW), Pathology (ZL, HW, XM), and Radiology (XC), Peking Union Medical College Hospital, Chinese Academy of Medical Sciences, No. 1 Shuaifuyuan Hutong of Dongcheng District, Beijing, PR China.

## Abstract

Multifocal cerebral gliomas (MCGs) represent approximately 10% of gliomas and are frequently mistaken as metastases of an unknown primary cancer site. Most MCGs are glioblastomas with <4 lesions supratentorially, and are lack of typical symptoms and special detections.

Through a rare MCG case, we aim to present this rarity and emphasize the need to correctly diagnose multiple intracranial lesions using a variety of diagnostic modalities to ensure that the patient receives proper treatment.

We present a case of multifocal cerebral anaplastic astrocytomas with a total of 8 lesions located in the left frontal lobe and invading the lateral ventricle, presenting with dysphasia and phantosmia. The disease course, including diagnosis and treatment, is presented and analyzed in detail. The pertinent literature is reviewed regarding this uncommon entity.

After an initial impression of brain metastasis from lung cancer because of the magnetic resonance imaging (MRI) resemblance and history of chronic bronchitis, we were able to use positron emission tomography (PET) and excisional biopsy to get the final diagnosis. After 10 months, the patient's overall condition deteriorated and succumbed to his disease.

MCGs are easy to be misdiagnosed as metastatic diseases. In addition to MRI, PET adds more biochemical and molecular information and is helpful in the differentiation. Although uncommon, if multiple lesions are present in various locations in the hemispheres, MCG should be kept in mind.

## INTRODUCTION

To be precise, MCGs could be classified into multicentric gliomas when there is no macroscopic or microscopic connection between the multiple brain tumors, and multifocal when there is evidence of microscopic connection or spread from a primary site. MCG can present with a clinical and radiological picture similar to that of metastatic disease, especially if there is a history of other cancers or clinical manifestation of other systems. Correct diagnosis can hardly be made without the help of advanced imaging technology and biopsy pathological examination. MCGs may either exhibit the same or different pathological patterns, among which, multiform glioblastomas are the most common.^2^ In only a few case reports, the MCGs exhibit oligodendroglioma, juvenile pilocytic astrocytoma (JPA) or cerebral anaplastic astrocytoma (CAA).^3,4^ The neurological appearance of MCG is nonspecific, but focal signs suggest the brain area that is malfunctioning. When the MCGs are resectable, surgery is recommended followed by radiotherapy and chemotherapy.^5^

Herein, we present a case of a 59-year-old woman suffering from multifocal CAA with a total of 8 lesions in the left frontal lobe and invading the lateral ventricle, presenting with dysphasia and phantosmia. The location and pathotype of the lesions and the patient's clinical features in this case are all unique. To the best of our knowledge, this is the first reported case of MCG presenting in this manner. Because the rarity of MCG often leads to misdiagnosis of intracranial metastatic tumors, abscesses, lymphoma, and demyelination, we also reviewed the latest literature on the imaging, diagnosis, and treatment of MCG.

## CASE REPORT

A 59 year-old Chinese female complained of olfactory hallucination for 1 month. She said she could sometimes perceive a smell of “baked sweet potato” or something like “a just died diplopod” in the complete absence of any physical odors. Moreover, her husband found she usually could not talk with him as fluently as before. She herself admitted having difficulty remembering words and memory deterioration. About 10 days after the onset, she complained of headache and vertigo (for 3 weeks before admission). She had no symptoms of unconsciousness, convulsion, epilepsy, or cognitive disorder and was not taking any medications. She noticed a slight weight loss despite a normal appetite. Except for a past medical history of chronic bronchitis, the patient was previously healthy. We did not identify any special circumstances regarding her family history and personal history related to her presentation. Upon neurological examination, the patient was in spirit. She could not remain focused on a simple task such as counting from 1 to 15. When asked about historical or verifiable personal events, her answers were incoherent and inconsistent and with lots of paraphasic errors. When asked to do manual or written alternating sequence tasks, her movements were uncoordinated. Her bilateral pupils were round, equal in size, and constricted visibly to light. Her muscle strength was 3/5 to 4/5 in the right extremities. Sensory function was normal. No apparent changes in superficial and deep tendon reflexes were observed. Babinski sign and Oppenheim sign at the right side were positive. Bilateral Hoffmann signs were positive. Pathological signs were absent.

**FIGURE 1:**
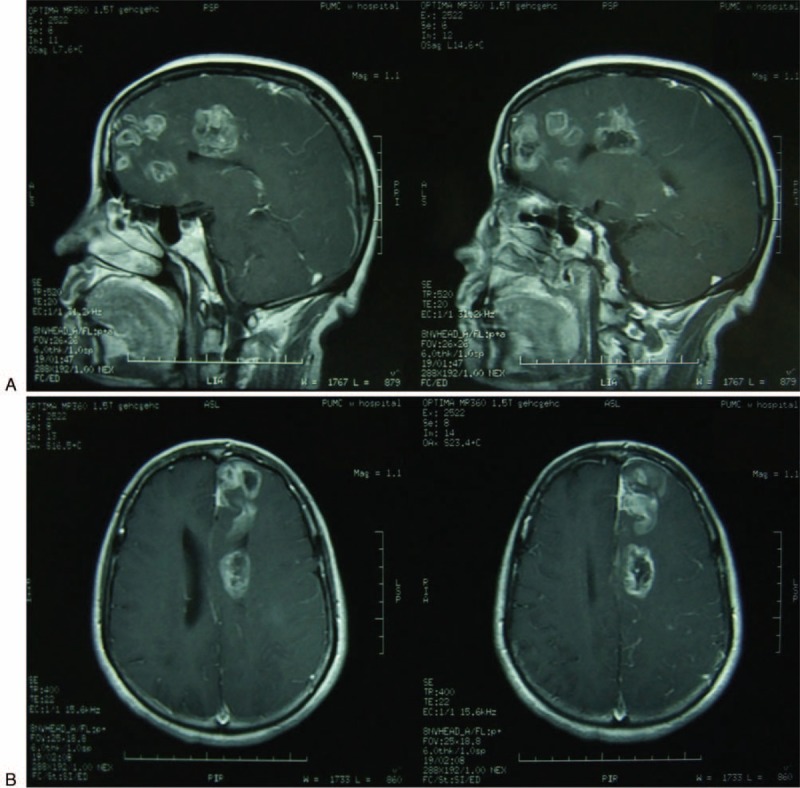
Magnetic resonance contrast-enhanced imaging of the patient's head revealed multifocal lesions in the left frontal lobe invading left lateral ventricle. These lesions had long T1 and long T2 signals with irregular margins and inhomogeneous enhancement.

Magnetic resonance imaging (MRI) of her head: bilateral cerebral hemispheres were symmetrical; multifocal spotted and patchy long T1 and long T2 signals were present in the left frontal lobe involving the falx, the centrum semiovale and corona radiata with decreased gray–white matter differentiation; fissures, sulci, and gyri of the corresponding brain area were not clear; the left lateral ventricle was also invaded by a much larger lesion with irregular tumor margins; T1-weighted image (T1WI) plus Gd-DTPA (gadolinium-diethylenetriamine pentaacetic acid) enhancement showed spotty circular-like or irregular rings of inhomogeneous enhancement and some had no intensification; there was light edema round the lesions, mild space occupying effect (Figure 1). Then, the patient underwent a lumbar puncture, showing an increased intracranial pressure at 228 mm Hg. Total cell count in cerebrospinal fluid (CSF) was 80 × 10^6^/L, WBC was 0, protein content was 560 mg/L. All the blood investigations were within normal limits. T/B cell subsets test: T 81.3%, T4 40.3%, T8 32.1%, T4/T8 1.26, B 10.2%.

Given the patient, as a smoker, had a history of chronic bronchitis, we initially suspected a diagnosis of lung cancer with brain metastasis. But except for some thickened bronchovascular shadows, chest X-ray did not show any suspicious nodules. The patient further underwent whole-body positron emission tomography (PET): multifocal irregular lesions with inhomogeneous high 18F-fluorodeoxyglucose (18F-FDG) uptake were detected in left frontal lobe near centerline. Some adjacent areas were also highly 18F-FDG avid (Figure 2  ). In other parts of the body, increased 18F-FDG uptake was not detected. Together, these images were representative of the features of a primary intracranial malignancy.

**FIGURE 2:**
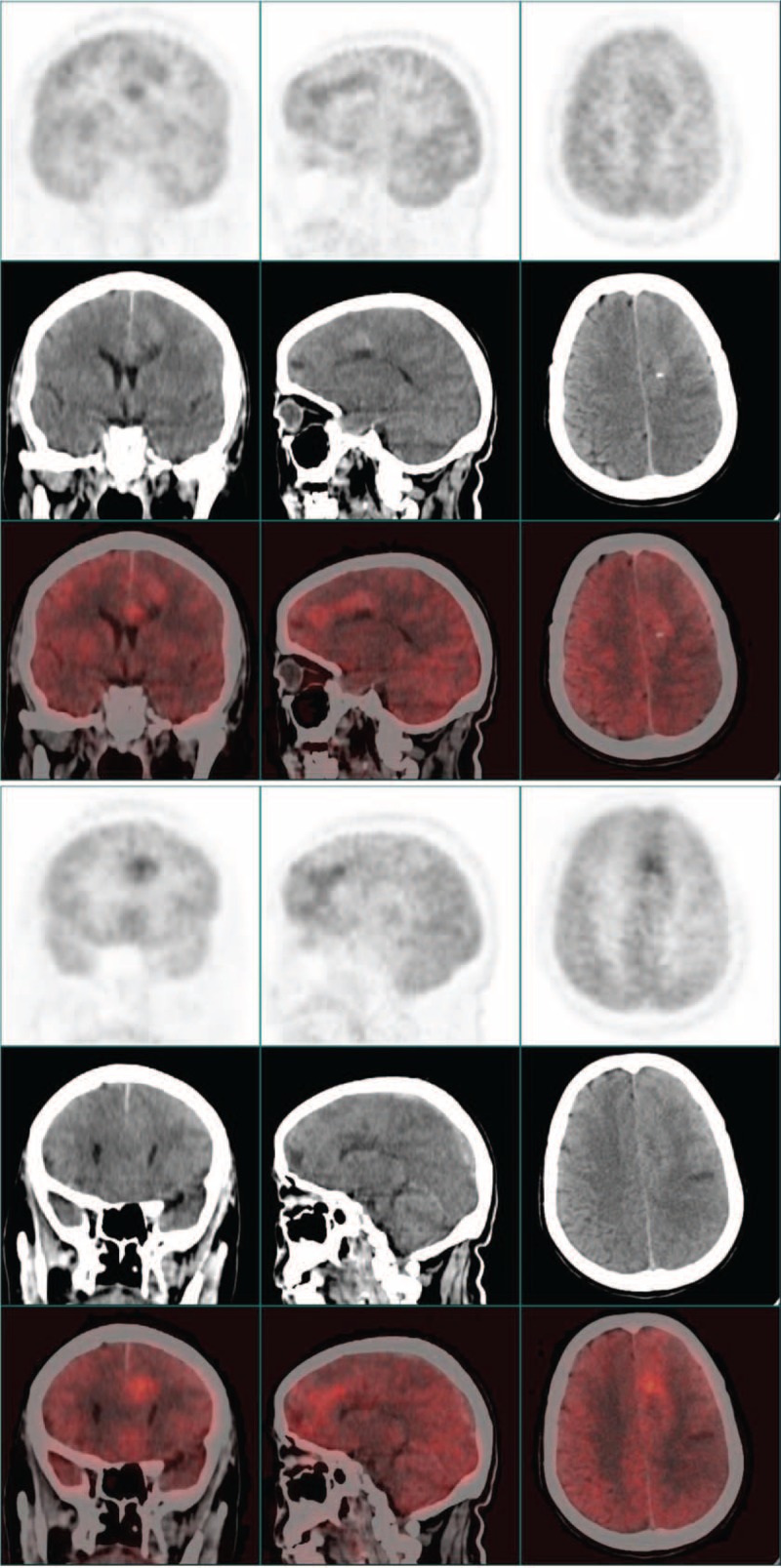
The figures of brain PET for this patient in different section levels. Multifocal irregular lesions with inhomogeneous high 18F-fluorodeoxyglucose (FDG) uptake were detected in left frontal lobe near centerline.

**FIGURE 2:**
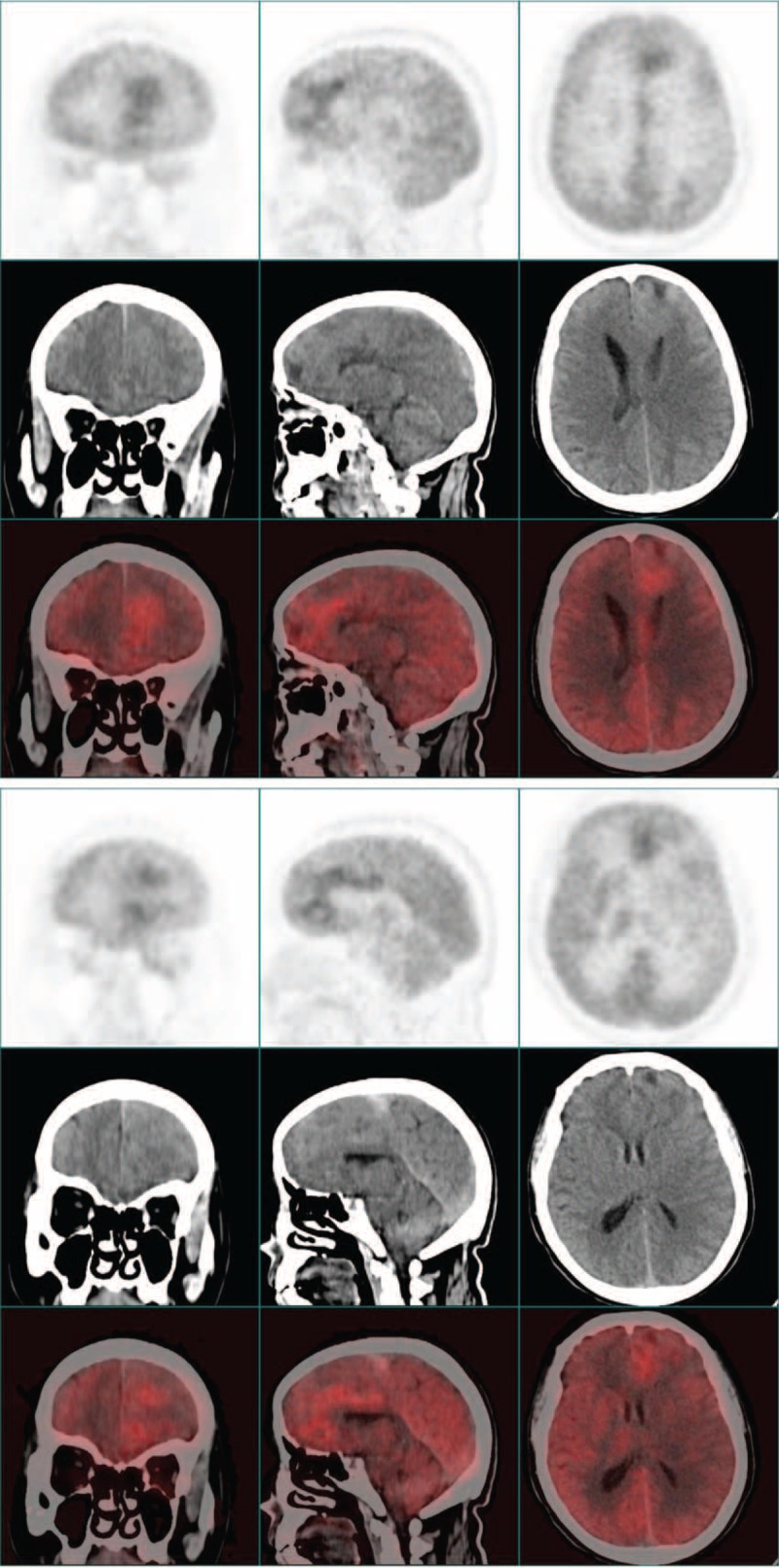
The figures of brain PET for this patient in different section levels. Multifocal irregular lesions with inhomogeneous high 18F-fluorodeoxyglucose (FDG) uptake were detected in left frontal lobe near centerline.

**FIGURE 2:**
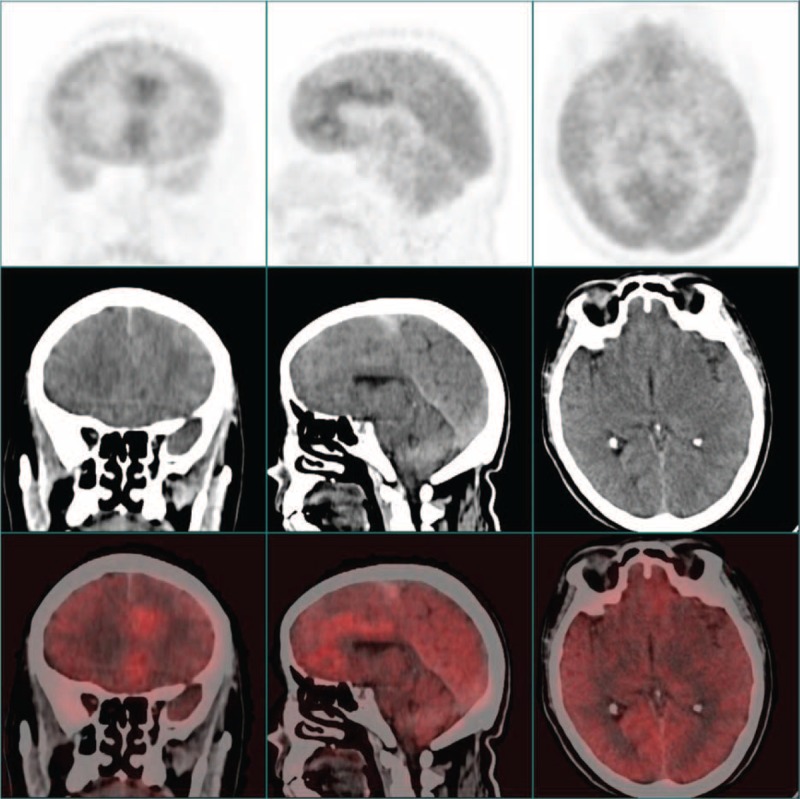
The figures of brain PET for this patient in different section levels. Multifocal irregular lesions with inhomogeneous high 18F-fluorodeoxyglucose (FDG) uptake were detected in left frontal lobe near centerline.

**FIGURE 3:**
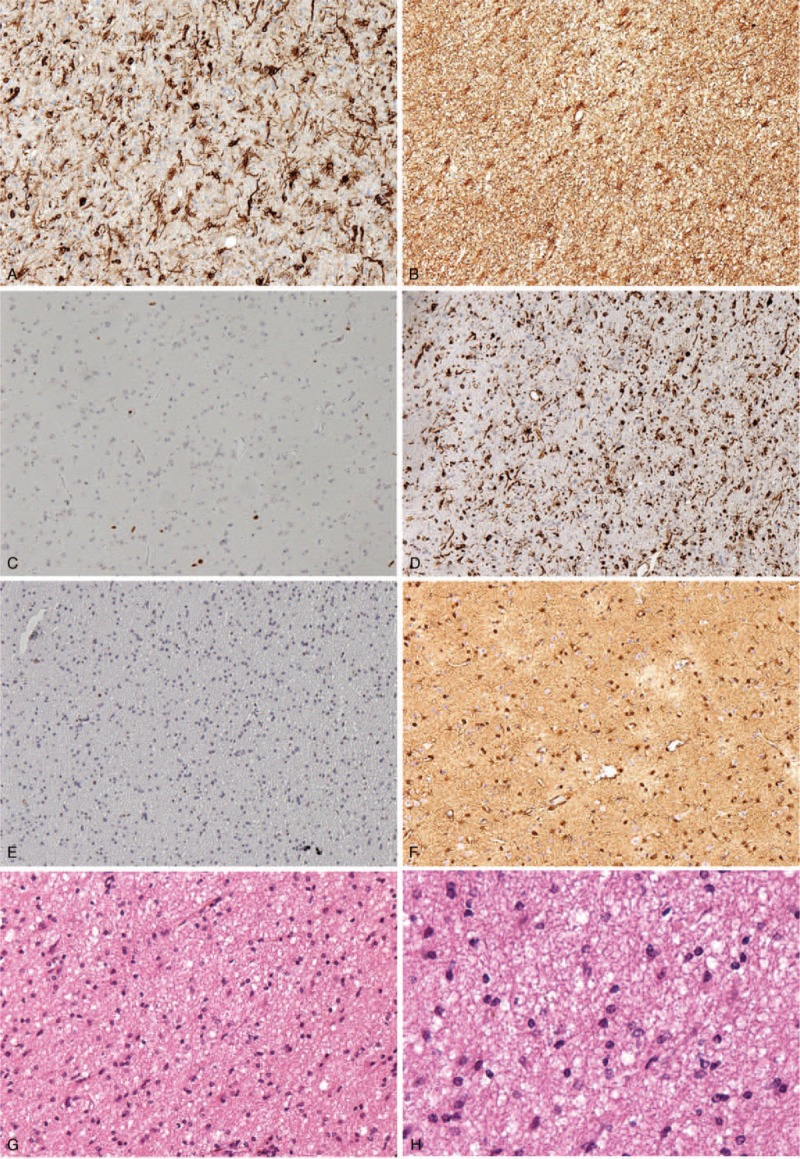
Immunohistochemical examination images. Epidermal growth factor receptor, glial fibrillary acidic protein, Nestin, P53, and S-100 were positive for this patient.

Then, we performed a craniotomy and tumor resection. The lesions were in white matter, with a soft, lobulated, light gray-pink appearance. Its texture was not homogeneous with some cystic changes. Fast frozen pathology of resected specimen during operation reported astrocytoma (World Health Organization [WHO] II–III). The paraffin histological examination confirmed the diagnosis as CAA. Immunohistochemical staining was positive for epidermal growth factor receptor, glial fibrillary acidic protein, Nestin, P53, S-100, CD34 (Figure 3); negative for epithelial membrane antigen, leukocyte common antigen, p53, and progesterone receptor. The proliferation index by Ki-67 exceeded 40%.

Postoperative MRI showed that the tumors were generally removed, and the left lateral ventricle was decompressed. The patient had an uneventful recovery with her headache and phantosmia much relieved, but her dysphasia never improved. Despite early neurological response to adjuvent chemotherapy with temozolomide (Stupp regimen), the patient's overall condition deteriorated about 10 months later secondary to the progression of her disease. She refused further treatment options and succumbed to her disease shortly thereafter. Autopsy was not performed.

## DISCUSSION

Described by Gower in 1896 for the first time,^6^ MCGs are a well-recognized but relatively uncommon entity, with a reported incidence of approximately 2 to 5% of total high-grade gliomas. There is a wide range in age at presentation of multiple gliomas, but the majority of patients are of middle–old age. No significant differences between sexes have been found. MCGs are often designated as multicentric or multifocal lesions. Multicentric gliomas are tumors arising independently in >1 site of the brain with absence of seeding along easily accessible routes. Multifocal gliomas are the result of dissemination of glioma cells from a primary focus to other parenchymal areas via the CSF, meninges or white matter tracts. According to the classifiation of Budka, multifocal gliomas are further grouped into 4 categories: diffuse, multiple, multicentric, and multiple-organ. MCGs can also be separated by time of occurrence. Present case is an example of multifocal CAA present at the same time.

The pathogenesis of multiple gliomas remains unknown. Many pathogenetic theories have been suggested to explain multiplicity. Zulch stated that multiple lesions are metastases from a primary focus via CSF or the white matter tracts. In the second theory, MCGs may arise from cells that, although not neoplastic in themselves, are nevertheless “primed” by an acquired or inherited genetic defect and scattered throughout the nervous system during development. The most recognized was Willis’ “ 2-step process” theory. In the initiation step, a large area of brain undergoes neoplastic transformation, thus more susceptible to neoplastic growth. In the promotion step, multiple areas of malignant transformation occur following various kinds of stimulation (biochemical, hormonal, mechanical, or viral), giving rise to multifocal glioma. A few genetic changes have been reported in MCGs, including *TP53* mutation, *BRCA-1* mutation, and deletion of chromosome 1p36, whereas TP53 mutation and PDGFR (platelet-derived growth factor receptor) overexpression represent early changes during low-grade glioma development, anaplastic progression is associated with pRB alteration and loss of heterozygosity (LOH) of 19q, further malignant progression to glioblastoma multiform including LOH 10q and mutations of *PTEN* gene. In a patient with multiple CAA, hereditary colorectal cancer, transcobalamin II deficiency, agenesis of the corpus callosum and mental retardation, a germline mutation of the *PMS2* gene was found. Furthermore, families with MCGs but without obvious connection to known tumor syndromes were described in several case reports and epidemiological studies.

MCGs may either exhibit the same or different histologies. Although glioblastomas are the most frequent pathotype, more benign glial neoplasms including astrocytoma and ependymoma have also been reported as multicentric. In the 51 cases reported by Kyritsis, there were 31 cases of glioblastomas followed by anaplastic gliomas (19 cases) and low-grade gliomas (1 case). In a case report, a 64-year-old female presented with a left frontal glioblastoma with astrocytic and neuronal differentiation and a temporal low-grade astrocytoma. In another case, a 43-year-old man presented with an oligodendroglioma and a JPA synchronously. A molecular analysis detected a deletion of chromosome 1p36 in the oligodendroglioma, but not in the JPA. A glioblastoma and a CAA plus a third unbiopsied glioma were reported in another patient. Other very rare cases of multifocality in low-grade gliomas have been reported in pleomorphic xanthoastrocytoma which has a favorable outcome with complete surgical resection. The histopathological features of multiple CAA include increased cellularity, distinct nuclear atypia and marked mitotic activity. Additional signs of anaplasia are nuclear inclusions, multinucleated cells, and abnormal mitoses. In our case also the histopathology was suggestive of CAA (WHO III). As a note of caution, however, histologic diagnosis and grading of astrocytomas is a somewhat subjective process.

The tumor locations are not fixed. In a retrospective study, supratentorial localization prevailed, but no hemispheric predilection was identified. According to Kyritsis, the parietal lobe was the most common sites (37%) followed by frontal lobe (28%) and temporal lobe (22%). Only a few cases were reported in thalamus, brain stem, occipital lobe or even spinal cord. Mostly, the number of tumors in an individual patient is not >4. According to a retrospective survey, among all MCG patients, 92% had 2 to 3 lesions and only <8% had 4 lesions. In our case, a total of 8 lesions presented as synchronous primary brain tumors in the same patient, which is very uncommon.

The clinical manifestations of are variable and non-characteristic, in relation to the extent of the lesions, including neurological focal signs (72%), epilepsy (40%), and symptoms of intracranial hypertension (68%) according to the above retrospective study. In our case, phantosmia and hypomnesis was 1 of the main complaints. Considering the lesions’ location, we speculate that the rhinencephalon (olfactory system) and part of the limbic system structures responsible for memory, were compressed and injured. This patient also presented with language impairment, which may be associated with lesions at dominant hemisphere.

In general, iso-to hypodense lesions are seen on CT with a more or less diffuse mass effect. On MRI, as in present case, MCGs are generally hypo- to isointense on T1WIs, hyperintense on T2-weighted and FLAIR images, and enhances strongly after contrast administration in a heterogeneous or ring-like manner. Meningeal or ventricular enhancement, suggestive of a possible way of dissemination, can be observed sometimes. Presence of mass effect, edema, and large tumor size will differentiation between the low and high grade, whereas the presence of necrosis or cyst with ring-enhancement are characteristics for glioblastomas. In general, MCGs usually involve white matter with indistinct tumor margins, inhomogeneous tumor enhancement, and mild edema, whereas brain metastases are often located near white–gray matter border with regular or variable borders, ring-shaped homogeneous enhancement, and obvious edema. However, considering the radiographic features are nonspecific, it is still difficult to make an accurate differentiation neuroradiologically. In contrast to MRI, clinical and experimental use of PET is expanding and allows quantitative assessment of brain tumors’ pathophysiology and biochemistry including glucose uptake, amino acid transport, protein/DNA synthesis, cell proliferation, angiogenesis, and oxygen tension. PET therefore provides different biochemical and molecular information about brain tumors when compared to histological methods or neuroradiological studies and is very helpful in the detection of MCGs detection, grading, differentiation, prognosis prediction, and treatment follow-up. In our case, the brain tumors were highly FDG avid, whereas no obvious hypermetabolism areas were detected elsewhere. Especially, as PET represents a novel technology for molecular imaging assays of metabolism and signal transduction to gene expression, reporter gene assays are used to trace the location and temporal level of expression of therapeutic and endogenous genes. Some differential points among MCGs, CAA, and brain metastasis are listed in Table 1 .

**TABLE 1:**
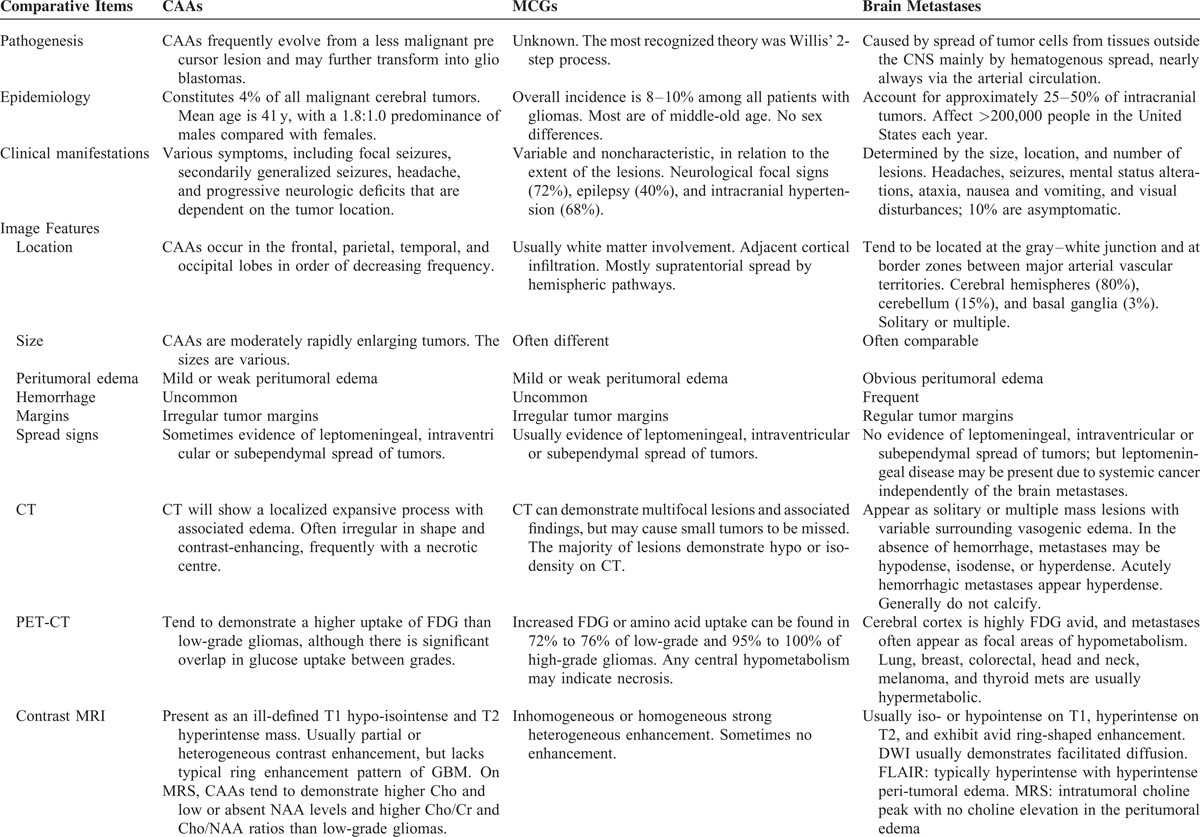
Differences Among CAA, MCG, and Brain Metastasis Tumor

**TABLE 1:**
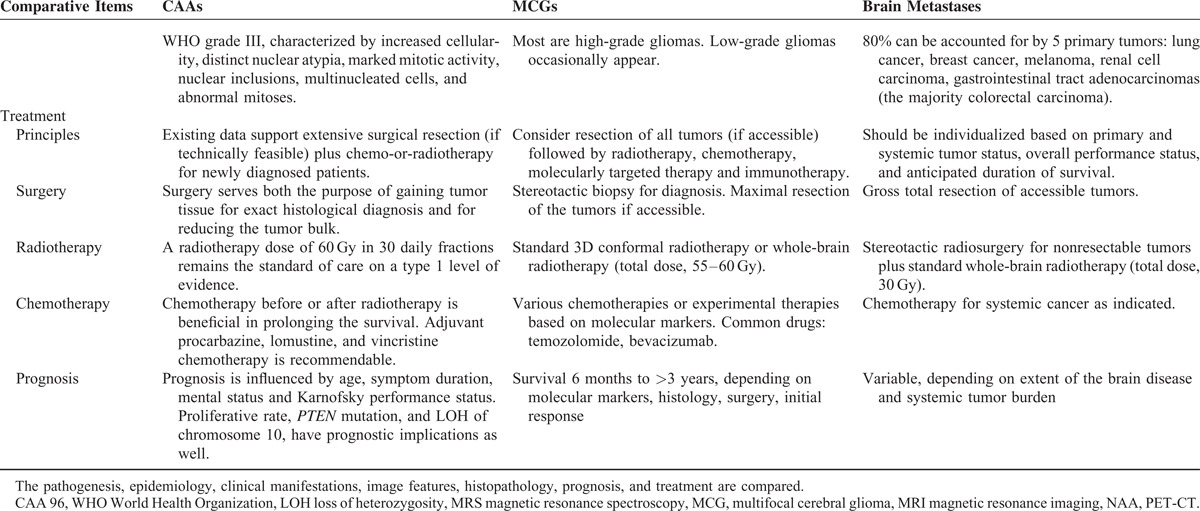
Differences Among CAA, MCG, and Brain Metastasis Tumor

A definite diagnosis of MCG should be confirmed by histopathological examination. So surgical intervention and tumor decompression not only have a significant impact on longer and better survival but also facilitate histopathological analysis. Moreover, adjuvant treatment may be more useful when the tumor bulk is already reduced. This statement stems from the fact that an aggressive surgical procedure for malignant gliomas with the resection of at least 90% of the tumor burden can result in increased survival duration for selected groups of patients. However, some other authors recommended biopsy as the fist step, believing that extensive resection increases the risk of hemorrhage and neurological deficits without influencing survival. Stereotactic biopsy has become a powerful and safe tool for providing tissue samples for diagnosis with minimal disruption of normal brain function, and plays a vital role in the management especially in cases where the lesions are located in sites inaccessible to surgery. In our case, we performed open surgical decompression of the larger lesions to improve the neurological deficits and also to get tissue for histological diagnosis. Apart from surgery, chemotherapy or radiation therapy may be considered.^2,5^ Although whole-brain radiotherapy is preferable, 3D conformal radiotherapy may be applied in order to avoid toxicity.^13^ The chemotherapy schedule of MCG is similar to common single glioma. Although the prognosis is associated with pathological types and molecular markers such as MGMT promoter methylation, IDH1 mutation, 1p/19q co-deletion,^11^ generally speaking, it remains unfavorable and most patients die within the first year after diagnosis as it is in our case.

## CONCLUSION

We report an uncommon case of multifocal CAA with a total of 8 lesions in the left frontal lobe and invading lateral ventricle, suffering from uncommon symptoms of dysphasia and phantosmia. MCGs are rare entities with the pathogenesis still unknown. Glioblastomas are the most frequent pathotype, but more benign ones such as the present case can also occur. MCGs are easy to be misdiagnosed as metastatic diseases according to radiological findings as it is in our case. In line with MRI, PET adds more biochemical and molecular information and is helpful in the differentiation. If multiple lesions are present in various locations in the hemispheres, MCG should be kept in mind. In such cases, advanced imageological examination or a biopsy is recommended for its differential diagnosis.

## UNCITED REFERENCES

^1,7–10,12^.
